# 20 Years of Telehealth in the Veterans Health Administration: Taking Stock of Our Past and Charting Our Future

**DOI:** 10.1007/s11606-024-08617-w

**Published:** 2024-02-20

**Authors:** Leonie Heyworth, Nilesh Shah, Kevin Galpin

**Affiliations:** Department of Veterans Affairs Central Office, Office of Connected Care/Telehealth, 810 Vermont Avenue, Washington, NW DC 20420 USA

## INTRODUCTION

**Figure 1:**
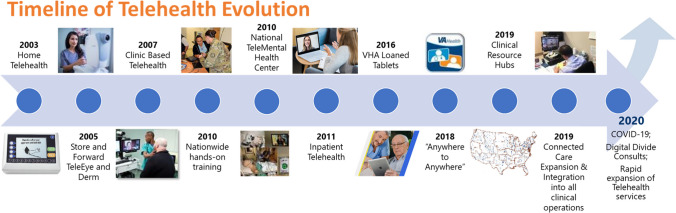
Timeline of VA telehealth evolution from 2003 to 2020 and beyond.

The Veterans Health Administration (VA) celebrated 20 years of telehealth services this April. Built with careful consideration over many years and tested during the pandemic, VA’s telehealth enterprise has proven to be robust, with approximately 40% of Veterans now receiving some kind of their VA care by telehealth. VA, a health system with almost 30% of its patients living in rural or highly rural areas, was uniquely positioned to benefit from resource-sharing on a national level. As technology platforms evolved and proliferated and use cases matured, VA also began to grasp a range of related barriers—from the authority to provide care by telehealth and the infrastructure actually needed to optimize and maximize the delivery of telehealth services, to knowing how best to prepare Veterans and their family members to use the technology. Figure 1 shows a 20-year timeline of selected developments in VA Telehealth. Here’s how we started and how it has evolved over two decades.

## OPERATIONALIZING CARE INTO THE HOME

VA’s inaugural “Home Telehealth” remote monitoring program^1^ launched in 2003, offering evidence-based chronic disease management for Veterans in their homes, facilitated by clinical care coordinators. This program was VA’s first nationally coordinated telehealth monitoring program. Biometric and condition-specific data gathered by devices in the home were transmitted back to VA and interpreted via expert-vetted disease management protocols where adjustments in medication, lifestyle, or further necessary work up could be assessed by a VA provider and communicated back to the Veteran for action. As this program grew in popularity, different evaluations demonstrated the program’s positive impact on various outcomes for patients with complex issues as well as notable cost-efficiencies.^2–5^

In February 2009, Congress passed the American Recovery and Reinvestmant Act, allocating as much as $27 billion over 10 years in support of the adoption of electronic health records (EHRs). The aim of this recovery plan, as described by President Obama to a joint session of Congress, was to “reduce errors, bring down costs, ensure privacy and save lives.” While federal agencies and the private sector responded to this new opportunity, VA, with its VistA electronic record having already been implemented in 1997, instead took a deep dive into building a patient-facing platform. The concept of patient-initiated communication with care teams, accessing data, and making requests—all electronically—represented another opportunity for Veterans to manage their care from outside the confines of the traditional office visit. By 2010, blue tents were populating the lobbies of VA facilities across the nation as part of outreach and promotional efforts focused on the VA online patient portal, MyHealth***e***Vet. As of October 2023, over 7.16 million Veterans have registered for a My HealtheVet account, over 260 million prescription refills have been requested, and over 210 million asynchronous secure messages between Veterans and VA clinical team members have been initiated.^6^

## A PUSH FOR TELEHEALTH EXPANSION: THE RISE OF SYNCHRONOUS AND ASYNCHRONOUS CLINICAL SERVICES

A nationally coordinated and centrally funded telehealth expansion effort kicked off in 2010 as one of VA Secretary Eric Shinseki’s focused priorities. This effort saw application of the newest synchronous technology and creation of the “telepresenter” concept, in which a technician served as the hands-on assistant to a remote provider and could facilitate a remote physical exam through critical actions such as stethoscope placement, assistance with exam maneuvers, and image capture. VA’s National Telehealth Program launched regional telehealth hands-on training nationwide. In these early days of clinical resource sharing at VA, outpatient clinics were the core of telehealth activity: patients could receive asynchronous services, such as eye screening and dermatologic lesion capture, and synchronous services from a VA provider in a different location. Though the advent of care by telehealth offered many exciting possibilities, barriers that VA faced during this phase included limited infrastructure for scheduling, bandwidth limitations, staffing limitations, fragmented and non-standardized processes to oversee qualifications and scope of remote providers, and de-centralized programs.

As a national healthcare system with a distinctly different reimbursement model from the private sector, the value proposition for telehealth at VA focused on enhancing accessibility, capacity, and quality of clinical care. The idea that clinical resource sharing could be executed at a national level began with VA’s first concept of a telehealth “hub”: the National TeleMental Health Center, established in 2010. This hub offered specialized mental health services to requesting sites via synchronous telehealth. The National TeleMental Health Center was a demonstration of VA’s ability to leverage clinical expertise from regions of the country where it existed, and enabling access to these services for sites in need. Standardizing access to any needed service by telehealth—whether for a Veteran living in a rural town needing a routine service, or Veterans living anywhere requiring highly specialized services—fueled the establishment of VA’s Clinical Resource Hubs (regionalized telehealth centers with dedicated administrative and clinical staff capable of efficiently delivering cross-facility care via telehealth) in 2019.

In 2015, recognizing rapid technologic advancements in telehealth technologies and the expanding portfolio of remote care, VA established the Office of Connected Care, uniting the Office of Telehealth Services and MyHealtheVet Online Patient Portal Program with a new Office of Connected Health, dedicated to mobile application development and patient-generated data. As the priorities of this new Office came into focus, one key priority was to support the expansion of acute care telehealth programs, such as TeleICU and TeleStroke, that could serve VA facilities across the country. As synchronous services grew from the outpatient to inpatient clinical environments, another telehealth modality—asynchronous services—was also on a growth trajectory. Asynchronous eye and dermatology services, which enable Veterans from any VA clinic to access these specialties through on-site image capture with remote interpretation by specialists across the country at a later time, provided 360,000 encounters from October 2022 to September 2023 with published literature demonstrating gains in access^7^ and noteworthy patient outcomes.^8^ Together, these multi-modality offerings further expanded VA’s vision of enabling standardized access to specialty care.

## EXPANDING CARE INTO THE HOME

As telehealth services blossomed within VA clinics and hospitals, VA began to build its enterprise solution for video telehealth into-the-home. To ensure equitable expansion of video into the home, in 2016, VA took early steps to bridge the digital divide through the distribution of loaned cellular enabled tablets to Veterans without a device or connectivity.^9^ In 2020, VA’s Office of Connected Care (OCC) formalized a “Digital Divide Consult,” through which social workers assisted Veterans with screening for eligibility to the federal Lifeline and Affordable Connectivity Programs as well as assisting with ordering a loaned device if needed. The Connected Devices Program added a digital skills and teaching component in 2021, where technicians call Veterans to orient them to their tablets and conduct a video test call. From an equity perspective, the Connected Devices Program has demonstrated improved continuity, better medication compliance, reduced visits to the ER among rural Veterans, and high satisfaction among participating Veterans.^10–12^

**Figure 2:**
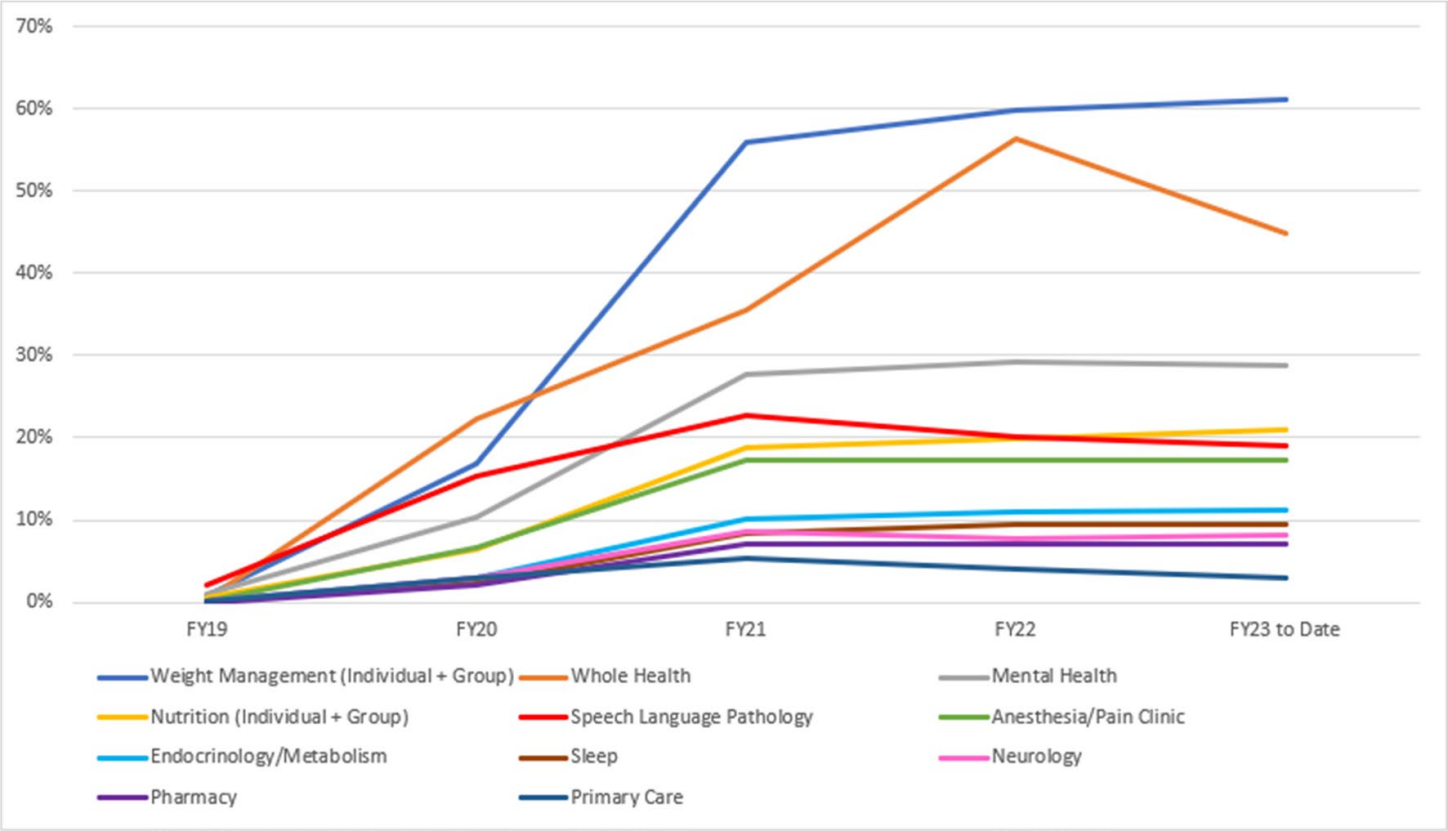
Video-to-home encounters as a percentage of total encounters across all modalities among Primary Care and the top ten clinical specialties delivering video telehealth at VA, FY 2019–23.

By 2017, VA had its first iteration of a video-to-home platform available for use, a capability which would enable Veterans to receive care on their own device, in their own homes or location of choice. Unique to VA’s approach to development of its video-to-home platform was customization, enabling enhanced features to manage clinical emergencies as an important example. Additional features intended to enhance user experience and safety were also incorporated over time, including a soon to be released “silent signal” alarm to alert remote providers that a patient may not be in a setting conducive to speaking openly about their health. Figure 2 shows the expansion of video visits to the home as a percentage of all VA encounters across 11 different specialties.

## THE CRITICAL ROLE OF VA POLICY AND LEGISLATION IN THE EXPANSION OF VA TELEHEALTH

With developments in technology that could now reach into Veterans’ homes, a critical barrier remained. To realize the full potential of telehealth across a national healthcare system, VA healthcare professionals needed the authority to deliver healthcare into non-federal spaces across state lines; however, no existing laws offered clear pre-emption of state law. In 2018, the Mission Act laid the groundwork for national resource sharing via telehealth through the “Anywhere to Anywhere” legislation and enabled VA to expand its telehealth infrastructure by way of a network of regional hubs, interconnected by standard processes with the flexibility of providing care within state, regionally or nationally. Following the passage of “Anywhere to Anywhere”, VA took an additional step forward to curate private spaces in broadband-poor areas for Veterans to receive VA telehealth services. Launched initially as a private-public partnership pilot, the Accessing Telehealth through Local Area Stations (ATLAS)^13^ program—allowing Veterans to receive care on Main Street, such as in a Veteran Service Organization post—brought together companies knowledgeable in matters related to high-speed internet, space design, and healthcare to create a concept that could further leverage VA’s “Anywhere to Anywhere” authorities. Additionally, to facilitate expansion of video services and to ensure that they remained accessible and affordable, VA entered into agreements with select major cellular carriers to minimize data charges for Veterans using VA’s video platform on their own devices.

## PANDEMIC EXPANSION

As stay-at-home orders rippled across the USA to control the spread of a new coronavirus, in 2020, VA issued guidance to defer routine in-person visits and escalate the use of virtual modalities for care. To accommodate the overnight surge in demand for virtual care, the video architecture was rapidly scaled to allow for what was to be over 3000% growth in video telehealth^14^ within VA over the next year. At the same time, new telework guidance, procurement and distribution of teleworking equipment, just-in-time trainings for clinical and administrative staff, and efforts to meaningfully allocate bandwidth at facilities in anticipation of a remote-working surge were underway. A national workgroup representing clinical specialties ranging from Urology to Nutrition and beyond met weekly to address feedback from frontline providers, articulate roles and responsibilities of clinical teams regarding the new roll-out of video, and set growth goals for all clinical areas. At the same time, VA’s Virtual Care Consortium of Research^15^ (Virtual Care CORE) was launched—an initiative dedicated to virtual care evaluation, quality and research. In its first 4 years supported by the Virtual Care CORE, OCC has funded 46 evaluation or quality improvement projects with a total budget of >$9.5 million.

Pandemic undertakings made way for progressive adoption and sustainment of video use. VA facilities embarked on a national initiative in 2021–2022 to improve experience in telehealth focused around high-impact “moments that matter” in a Veteran’s telehealth journey—with associated rises in experience and trust scores, in what has become VA’s relentless pursuit of positive patient experience with telehealth.

## THE FUTURE

In 2019, VA formalized a blueprint for enterprise telehealth services: specific tactics to build VA’s digital front door for Veterans and a roadmap for the development and dissemination of virtual care tools. This blueprint included key proposed policies designed for optimal clinical resource sharing via telehealth, including such goals as a new regional approach to credentialing and privileging to enable workforce oversight, a telework policy, and further efforts to bridge the digital divide that prioritized equity and access.

At a time when so many Veterans receive some portion of their care by telehealth and Veteran preferences for telehealth remain as popular among experienced users as during the height of the COVID-19 pandemic, VA continues to build upon pre-pandemic foundations in all aspects of virtual care. With anticipated further expansion ahead, maximizing quality and equity remain guiding principles. Integrating patient-generated health data from personal devices such as wearables and disseminating technologies that facilitate access to services for more Veterans present great potential to expand reach and engagement. New opportunities to enhance the standard of VA care through EHR capacity-building that will leverage resources on a national scale and national standardization of telehealth metrics have transformative potential for our healthcare system. With other emerging technologies like artificial intelligence on the horizon, the future of virtual care has yet to be defined. Understanding its role in healthcare—particularly how it can augment traditional offices visits in a safe and patient-centered fashion, will be paramount.

## References

[1] Understanding Remote Patient Monitoring | Veterans Affairs. Accessed November 28, 2023. http://www.veteranshealthlibrary.va.gov/RelatedItems/142,41534_VA

[2] Darkins A, Ryan P, Kobb R (2008). Care Coordination/Home Telehealth: the systematic implementation of health informatics, home telehealth, and disease management to support the care of veteran patients with chronic conditions. Telemed J E Health..

[3] Chumbler NR, Chuang HC, Wu SS (2009). Mortality risk for diabetes patients in a care coordination, home-telehealth programme. J Telemed Telecare..

[4] Darkins A, Kendall S, Edmonson E, Young M, Stressel P (2015). Reduced cost and mortality using home telehealth to promote self-management of complex chronic conditions: a retrospective matched cohort study of 4,999 veteran patients. Telemed J E Health..

[5] Noel HC, Vogel DC, Erdos JJ, Cornwall D, Levin F (2004). Home telehealth reduces healthcare costs. Telemed J E Health..

[6] My HealtheVet Statistics - My HealtheVet Product. Accessed November 28, 2023. https://vaww.va.gov/MYHEALTHEVET/statistics.asp

[7] Peracca SB, Jackson GL, Lamkin RP (2021). Implementing Teledermatology for Rural Veterans: An Evaluation Using the RE-AIM Framework. Telemedicine and e-Health..

[8] Ashrafzadeh S, Gundlach BS, Tsui I (2021). Implementation of Teleretinal Screening Using Optical Coherence Tomography in the Veterans Health Administration. Telemedicine and e-Health..

[9] Zulman DM, Wong EP, Slightam C (2019). Making connections: nationwide implementation of video telehealth tablets to address access barriers in veterans. JAMIA Open..

[10] Jacobs JC, Blonigen DM, Kimerling R (2019). Increasing Mental Health Care Access, Continuity, and Efficiency for Veterans Through Telehealth With Video Tablets. Psychiatr Serv..

[11] Gujral K, Van Campen J, Jacobs J, Kimerling R, Blonigen D, Zulman DM (2022). Mental Health Service Use, Suicide Behavior, and Emergency Department Visits Among Rural US Veterans Who Received Video-Enabled Tablets During the COVID-19 Pandemic. JAMA Netw Open..

[12] Slightam C, Gregory AJ, Hu J (2020). Patient Perceptions of Video Visits Using Veterans Affairs Telehealth Tablets: Survey Study. J Med Internet Res..

[13] ATLAS | Connected Care. Accessed November 28, 2023. https://connectedcare.va.gov/partners/atlas

[14] **Heyworth L, Kirsh S, Zulman D, Ferguson JM, Kizer KW.** Expanding Access through Virtual Care: The VA’s Early Experience with Covid-19. Catalyst non-issue content. 2020;1(4). 10.1056/CAT.20.0327

[15] Virtual Care Consortium of Research (VC CORE). Published June 29, 2023. Accessed November 28, 2023. https://www.hsrd.research.va.gov/centers/core/virtual_care/

